# Diagnostic Role of Colon Capsule Endoscopy in Patients with Optimal Colon Cleaning

**DOI:** 10.1155/2016/2738208

**Published:** 2016-03-15

**Authors:** Ümit Akyüz, Yusuf Yılmaz, Ali Tüzün İnce, Bülent Kaya, Cengiz Pata

**Affiliations:** ^1^Department of Gastroenterology, Yeditepe University, Kozyatağı, 34752 Istanbul, Turkey; ^2^Department of Gastroenterology, Marmara University, Maltepe, 81090 Istanbul, Turkey; ^3^Department of Gastroenterology, Bezm-i Alem University, Fatih, 34590 Istanbul, Turkey; ^4^Department of General Surgery, Fatih Sultan Mehmet Training and Research Hospital, Ataşehir, 34752 Istanbul, Turkey

## Abstract

*Background*. Colon capsule endoscopy (CCE) is a diagnostic test with relatively rare usage. In this study, we aimed to evaluate both the optimal cleaning regimen for CCE and the diagnostic value of test in the study group.* Methods*. A total of 62 patients were enrolled in this study. In the first step, 3 different colon preparing regimens were given to 30 patients [*Group A*: 3 days of liquid diet, sodium phosphate (NaP) (90 mL), and NaP enema;* Group B*: 3 days of liquid diet, 4 L of polyethylene glycol (PEG), and metoclopramide;* Group C*: 3 days of liquid diet, 4 L of PEG, NaP (45 mL), and bisacodyl after capsule ingestion] (10 patients in each group). The other consecutive 32 patients were cleaned with the best regimen which was NaP + PEG and CCE was performed. The results of CCE were controlled with colonoscopy in 28 patients.* Results*. Group C had the highest cleaning score, compared with the other groups (2.2 ± 0.4 versus 2.7 ± 0.4 versus 3.7 ± 0.4, *p* value = 0.000). The CCE findings were as follows in 28 patients who were also examined with colonoscopy: polyp (range: 5–10 mm) in 6 patients, internal hemorrhoids in 3 patients, angiodysplasia in 1 patient, diverticula in 1 patient, and ulcerative colitis in 1 patient. The sensitivity, specificity, PPV, and NPV of CCE were 100%, 92%, 93%, and 100%, respectively.* Conclusions*. Low dosage NaP combined with PEG provides optimal bowel preparation for CCE. CCE appears to be a highly sensitive diagnostic modality for detecting colonic pathologies.

## 1. Introduction

Capsule endoscopy is the gold standard noninvasive technique for small bowel pathologies, especially for obscured gastrointestinal bleeding. As a result of advances in technology, colon capsule endoscopy (CCE) was developed in 2006 [1–3]. Conventional colonoscopy is the gold standard for examination of the colon. However, the rate of serious adverse events is 2.8 per 1000 procedures including bleeding, perforation, and infection and these complications may increase during therapeutic interventions [4–8]. Clinical use of CCE is not so clear and there are a limited number of studies. The sensitivity and specificity rates of CCE for detecting any size of polyp were 73% and 89%, respectively. Cleanliness of the colon was also found to be the most important factor for increasing the diagnostic value of CCE [9]. However, an optimal cleaning regimen for CCE was not established [9–13]. In this study, we aimed to evaluate both the optimal cleaning regimen for CCE and the diagnostic value of test in the study group.

## 2. Material and Methods

### 2.1. Patients

From January 2010 to December 2012, a total of 62 patients were enrolled in this prospective study. The patients with gastrointestinal dysmotility, suspected or known bowel strictures, pregnancy, abdominal surgery in the past 6 months, and life-threatening condition were excluded. All subjects included in this study signed the written informed consent prior to the study. The local ethical committee approved this study.

**Figure 1:**
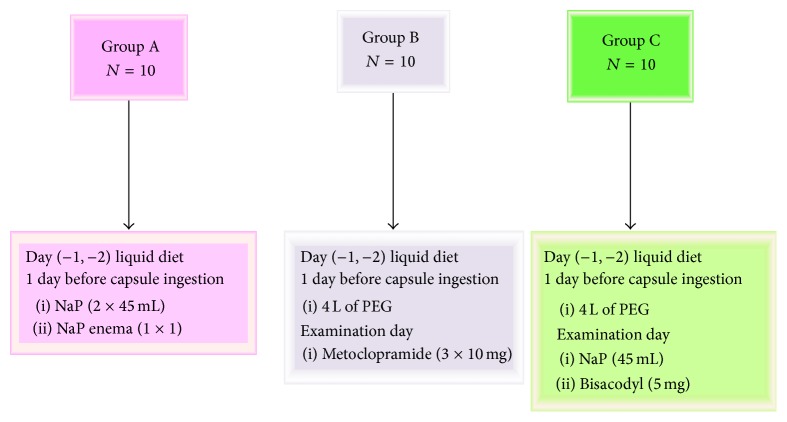
Cleaning regimens of this study (PEG: polyethylene glycol; NaP: sodium phosphate).

To evaluate the optimal colon cleaning, 3 different colon preparation regimens were given to 30 patients which were divided into 3 groups:

 
*Group A*: 3 days of liquid diet, sodium phosphate (NaP) (45 mL) (second day: 15^00^ 1 × 1, 18^00^ 1 × 1), and NaP enema (second day: 20^00^).
 
*Group B*: 3 days of liquid diet, 4 L of polyethylene glycol (PEG) (second day: 16^00^), and metoclopramide (10 mg) (third day: 9^00^, 12^00^, and 16^00^).
 
*Group C*: 3 days of liquid diet, 4 L of PEG (second day: 16^00^), NaP (45 mL) (third day: 9^00^), and bisacodyl (5 mg) after capsule ingestion (Figure 1).

Cleaning was evaluated by scaled observations during the examination (perfect = 4; good = 3; intermediate = 2; poor = 1) (perfect: no feces; good: very small amount of feces or dark fluid; intermediate: feces/dark fluid present preventing total examination; poor: large amount of feces). After finding an optimal colon preparation regimen, all the other 32 patients cleaned with this regimen.

### 2.2. Endoscopic Procedures

A second-generation colon capsule endoscopy, Pillcam Colon Capsule-2 (Given Imaging Ltd., Yoqneam, Israel), was used for the CCE. The capsule battery life was at least 10 hours. CCE analyses were done using RAPID software.

The conventional colonoscopy (Fujinon 450 system, Japan) was performed on the same day or the next day in the patients who accepted this procedure. Two experienced endoscopists performed the colonoscopy and analyzed the capsule endoscopy separately. They were blinded to the results of CCE. All the positive findings were recorded with their location and size.

A total of 28 patients had both colonoscopy and CCE. The other patients did not accept colonoscopy. Therefore, we evaluated the results of these 28 patients to determine the diagnostic value of CCE.

### 2.3. Statistical Methods

The sensitivity, specificity, positive predictive value (PPV), and negative predictive value (NPV) of the CCE versus conventional colonoscopy were evaluated. A second descriptive analysis was done by SPSS 15. Nonparametric Mann-Whitney *U* and Kruskal-Wallis tests were used where appropriate. A *p* value of 0.05 or less was considered to be statistically significant.

## 3. Results

The mean age of patients was 55.8 ± 8.2 years. There were 21 male and 41 female patients. For colon preparation, Group C had the highest cleaning score compared with the other groups (*p* = 0.001). Cleaning scores were shown in Table 1. The CCE excretion rate was 100% and the whole colon was examined in all patients in Group C. Mean gastric transit time (GTT), small bowel transit time (SBTT), and colon transit time (CTT) were 57 ± 31 min, 52 ± 21 min, and 30 ± 15 min; 2.3 ± 0.73 h, 3 ± 0.9 h, and 2.7 ± 0.6 h; and 6.8 ± 2.3 h, 7.2 ± 2.8 h, and 4.2 ± 1.3 h in Groups A, B, and C, respectively. Mean GTT, SBTT, and CTT were similar in Groups A and B (*p* > 0.05). Mean GTT and CTT were shorter in Group C than in Group B (*p* = 0.023 and *p* = 0.019, resp.) and Group A (*p* = 0.009 and *p* = 0.043, resp.) (Table 1).

**Figure 2:**
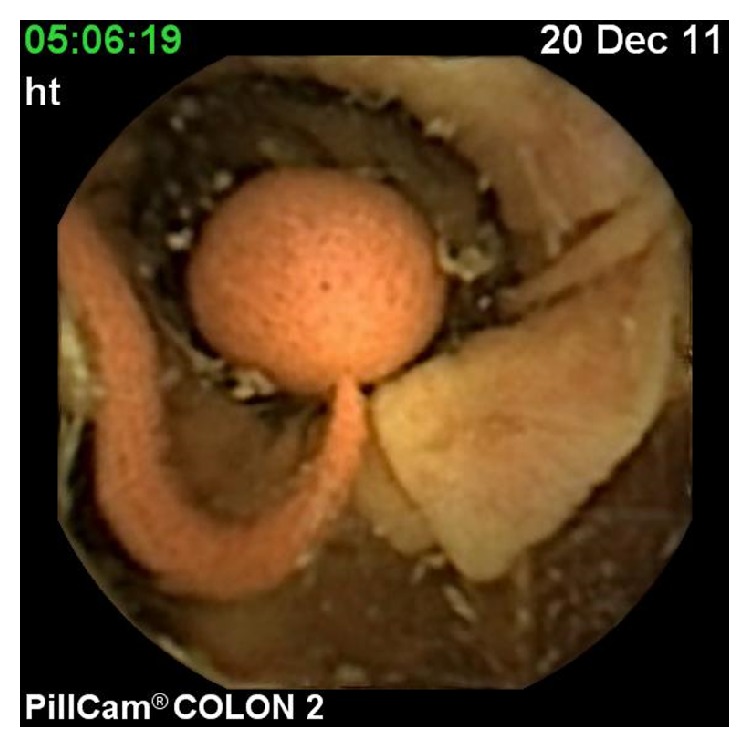
Image of pedunculated polyp in transverse colon in CCE.

**Figure 3:**
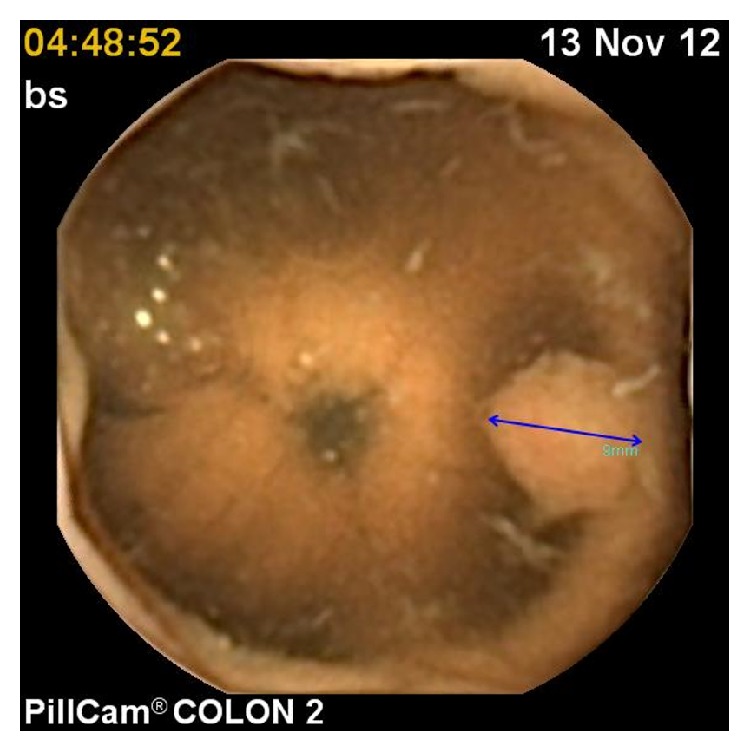
Image of polyp (blue arrow) in transverse colon.

**Figure 4:**
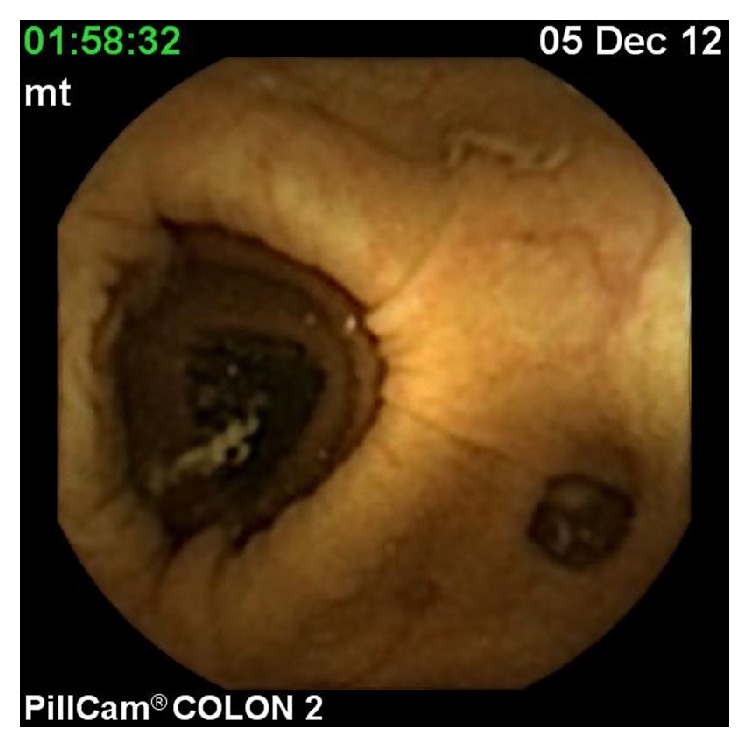
Diverticulosis coli.

The CCE findings were as follows in 28 patients who were also examined with colonoscopy: polyp (range: 5–10 mm) in 6 patients (Figures 2 and 3), internal hemorrhoids in 3 patients, angiodysplasia in 1 patient, diverticula in 1 patient (Figure 4), and ulcerative colitis in 1 patient (Table 2). The remaining patients were normal. Additional polyps were detected in 2 patients using colonoscopy. In one patient, a polyp was reported during CCE that was not detected with colonoscopy. CCE sensitivity, specificity, positive predictive value (PPV), and negative predictive value (NPV) were analyzed. The sensitivity, specificity, PPV, and NPV of CCE for the diagnosis were 100%, 92%, 93%, and 100%, respectively. No adverse events occurred during the preparation and/or examination periods.

## 4. Discussion

Colon capsule endoscopy is a new technique for examining the colon. However, the inability to wash the mucosa is an important disadvantage of CCE. The main problem with this examination is bowel preparation. There is no perfect regimen for all patients. We aimed to determine the most appropriate cleaning regimen for CCE in routine practice. Therefore, in the first step of our study, we compared 3 different cleaning regimens. Our results showed that the low dose booster of NaP on the day of examination and 4 L of PEG solution on the day prior to examination was the best cleaning regimen. Most studies in the literature aimed to determine the optimal cleaning regimen for CCE. Multiple laxatives and drug combinations with high doses were used in these studies [2, 3, 9, 11]. These regimens included prokinetics and additional laxative boosters after capsule ingestion to obtain excellent cleaning of colon and to examine all colon segments during the limited battery time (approximately 10 hours). Excellent cleaning rate was approximately 65–80% in all of these studies. However, these combinations are not easy to use in routine practice. For example, Tegaserod is not allowed in routine practice anymore because of serious life-threatening cardiac adverse events. NaP should also be used carefully in patients who have a low glomerular filtration rate and geriatric groups. Renal function impairment is a dangerous adverse effect of NaP. Sieg et al. [10] showed that if NaP is removed, the excretion rate drops. We wanted to use the lowest dose of NaP possible in order to prevent the potential adverse effects. There are few studies that have compared different cleaning regimens. We compared the effects of three different preparation regimens and showed that a combination of low dose NaP and 4 L of PEG solution was the optimal regimen for colon preparation for CCE. Preparation of the left colon and rectosigmoid region was also optimal with our regimen. Eliakim et al. [11] used a split dose of PEG solution (2 litres in the evening before and 2 litres on the day of capsule ingestion) with a low dose (45 mL) of NaP boosters. Using this regimen, they obtained excellent cleaning in 78% of patients and excretion rate within 8 h in 81% of capsule colonoscopies. Spada et al. [13] compared the efficacy of standard (clear liquid diet, 4 L of PEG, and 1 or 2 NaP boosters) and modified (low-residue diet; boosters were substituted by 1 or 2 PEG boosters and 4 senna tablets) cleaning regimens. They used PEG booster instead of NaP booster. Diagnostic yield (63% versus 87%; *p* = NS) and cleaning (35% versus 53%; *p* = NS) were not different between standard and modified regimens. However, the standard regimen capsule excretion rate was higher (100%) than the modified regimen (75%). NaP is able to achieve more vigorous activation of the peristalsis compared with PEG, resulting in a faster transit time and a higher completion rate of CCE examinations. We did not need a higher NaP booster, because we showed that a low dose (45 mL) of NaP booster was effective for obtaining excellent cleaning and providing for total examination of the colon. NaP is not preferred in most centers due to the risk of adverse effects. Studies are underway using low volume PEG and bisacodyl as an engine but as yet no results are available [14]. Kakugawa et al. [15] showed that reduced volume of bowel preparation regimen (did not drink PEG solution the day before the capsule procedure, 2 L PEG during the procedure day) was as effective as the commonly used higher volume (drank 2 L of PEG the day before the procedure and 1 L of PEG solution during the procedure day) method for colon capsule endoscopy. Hartmann et al. [16] used NaP-free PEG bowel preparation (PEG + ascorbic acid) for capsule colonoscopy. Good or excellent cleaning was obtained in 83% of patients.

Diagnostic role of CCE is still debated. Another aim of this study was to investigate the diagnostic accuracy of CCE in colonic pathologies. The diagnostic accuracy of CCE was found to be excellent in this study. To date, CCE was accepted as a feasible and reliable technique to detect colonic lesions, such as polyps and tumor in the literature. As an important colonic pathology diagnosis of inflammatory bowel disease with CCE was not recommended because it requires biopsy and histological confirmation. But it may be a useful tool to guide therapy, especially for detecting mucosal healing. Rokkas et al. [17] reported per-patient CCE sensitivity of 73% and specificity of 89% for any polyp in their meta-analysis. CCE can be used to screen for polyps and colorectal cancer with the technological improvements and better bowel preparation. One of the important indications of CCE is in patients with previous incomplete colonoscopy. Many studies showed that CCE was able to complement a previous incomplete colonoscopy. It may be successful to visualize the colonic segments not seen by previous incomplete conventional colonoscopy. It may detect polyps smaller than 6 mm and when compared to colonoscopy it is less invasive. Although CCE is specifically used for the colon examination, it can also show the small bowel. If the capsule is activated before the ingestion, the whole gastrointestinal system can be investigated. In our study, CCE was associated with high sensitivity, specificity, positive predictive value (PPV), and negative predictive value (NPV).

In conclusion, in this study, low dosage NaP combined with PEG provided optimal bowel preparation for CCE and CCE was highly sensitive in detecting the colonic pathologies.

## Figures and Tables

**Table 1:** Cleaning scores and imaged segments of colon by CCE.

	Jejunum-ileum cleaning scores mean	Colon cleaning scores mean	Imaging of right colon *N*	Imaging of total colon *N*
Group A	4	2.2 ± 0.4	9	5
Group B	4	2.7 ± 0.4	10	6
Group C	4	3.7 ± 0.4	10	10

**Table 2:** CCE and colonoscopic findings of patients.

*N* = 28	CCE	Colonoscopy
Normal	16	15
Polyp	6	7
Internal hemorrhoids	3	3
Angiodysplasia	1	1
Diverticula	1	1
Ulcerative colitis	1	1
